# The Effect of Thyroid Surgery on the Accuracy of Palpation-Based Cricothyroid Membrane Identification in Female Patients: A Prospective Observational Cohort Study

**DOI:** 10.3390/medicina60030471

**Published:** 2024-03-13

**Authors:** Jaesik Park, A Rim Yang, Hyunji Lee, Kwangsoon Kim, Min Suk Chae

**Affiliations:** 1 Department of Anesthesiology and Pain Medicine, Seoul St. Mary’s Hospital, College of Medicine, The Catholic University of Korea, Seoul 06591, Republic of Korea; 2 Department of Anesthesiology and Pain Medicine, Uijeongbu St. Mary’s Hospital, College of Medicine, The Catholic University of Korea, Seoul 06591, Republic of Korea; kallikalli0502@gmail.com; 3 Department of Anesthesiology and Pain Medicine, Bucheon St. Mary’s Hospital, College of Medicine, The Catholic University of Korea, Seoul 06591, Republic of Korea; auswl96@naver.com; 4 Department of Surgery, Seoul St. Mary’s Hospital, College of Medicine, The Catholic University of Korea, Seoul 06591, Republic of Korea; noar99@naver.com

**Keywords:** cricothyroid membrane, thyroid surgery, female

## Abstract

*Background and Objectives*: This study examined how a history of thyroid surgery impacts the precision of cricothyroid membrane (CTM) identification through palpation (validated by ultrasound) in female patients visiting the operating room for surgeries unrelated to neck procedures. *Materials and Methods*: This prospective observational cohort study enrolled adult female patients undergoing elective non-neck surgery, dividing them into control (no thyroid surgery history; *n* = 40) and experimental (with thyroid surgery history; *n* = 40) groups. CTM identification was performed by palpation and confirmed via ultrasound. *Results*: There were no significant differences between two groups in the demographic characteristics of the patients. The success rate and accuracy of CTM identification through palpation were significantly higher in the control group compared to the experimental group (90% vs. 42.5%, respectively; *p* < 0.001). For female patients with a history of thyroid surgery, the sensitivity of successful CTM palpation was 42.5%, and the specificity was 10%. These figures are based on the calculated true positives (17), false positives (36), true negatives (4), and false negatives (23). *Conclusions*: Thyroid surgery history in female patients may hinder the accurate palpation-based identification of the CTM, suggesting a need for enhanced clinical practices and considerations during airway management training.

## 1. Introduction

Accurately identifying the cricothyroid membrane (CTM) and directly accessing the trachea are necessary for emergency front-of-neck access (eFONA) during airway management. Emergency situations such as “cannot ventilate, cannot oxygenate” are an indication for eFONA. The requirement for a cricothyroidotomy may emerge in the operating room, and has a high rate of failure [1,2]. Among the many factors involved in the high failure rate, misidentifying the CTM via external palpation is important. The CTM identification success rate by palpation is approximately 50% in patients without neck pathology [3,4]. The success rate reported in the literature ranges from 19% to 71% in female patients [1]. Misidentifying the CTM and inadvertently placing a needle through it can lead to oxygenation failure or a lethal airway injury [5]. Reducing the risk of incorrect identification of the CTM may be the first step to ensuring an intact airway in a patient with a neck pathology or surgical injury. Identifying the CTM can be more challenging in patients with poorly defined neck markers, such as those who are obese or female. Females have less prominent neck landmarks than males, who have an obvious laryngeal prominence that guides the identification of the CTM by palpation. Many studies have shown that female sex is a risk factor for misidentification of the CTM [6].

As of now, the study related to the association between a history of thyroid surgery and CTM palpation identification has not been established. However, thyroid surgery can potentially impact the palpation of the cricothyroid membrane (CTM) due to anatomical changes and tissue healing post-operation. The surgery involves a curved skin incision made well above the sternum, which is then extended laterally towards the sternocleidomastoid muscles. This incision, followed by the elevation of skin flaps and the strategic retraction of the strap muscles, is meticulously conducted to minimize tissue trauma and preserve structural integrity. However, even with such precision, the dissection and retraction required to expose the thyroid gland can lead to postoperative scarring and altered anatomical landmarks. These changes can sometimes result in a thicker or less pliable anterior neck tissue over time, which may obscure the CTM, making it more challenging to palpate. Furthermore, if the strap muscles are divided during surgery for better access—typically in cases of large or vascular goiters—this can lead to additional scar tissue formation. The subsequent healing process might alter the normal palpable cues used to locate the CTM. Therefore, any thyroid surgery has the potential to complicate the subsequent identification of the CTM by altering the usual landmarks and possibly introducing scar tissue, which can change the tactile feel of the neck anatomy during palpation [5,7,8].

This study examined how a history of thyroid surgery impacts the precision of cricothyroid membrane (CTM) identification through palpation (validated by ultrasound) in female patients visiting the operating room for surgeries unrelated to neck procedures.

## 2. Patients and Methods

### 2.1. Ethical Considerations

This prospective observational cohort study was approved by the Institutional Review Board of Seoul St. Mary’s Hospital Ethics Committee (approval number: KC20OISI0835) on 3 November 2020, and was performed according to the principles of the Declaration of Helsinki. The trial was registered at the Clinical Research Information Service, Republic of Korea (approval number: KCT0005603). Written informed consent was obtained from all patients, who were enrolled in the study between November 2020 and April 2021. The study adhered to the Strengthening the Reporting of Observational Studies in Epidemiology (STROBE) guidelines.

### 2.2. Study Population

**Figure 1:**
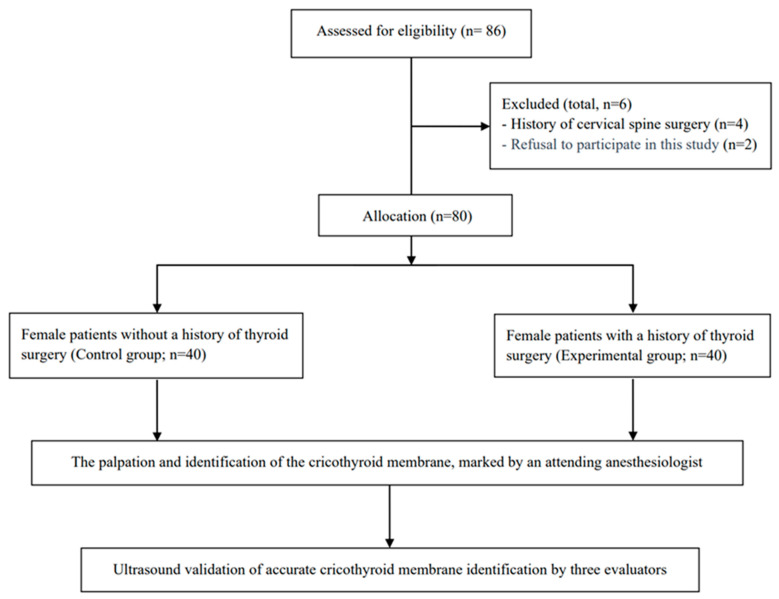
Study flow diagram.

Data were collected from 86 patients between November 2020 and April 2021 at Seoul St. Mary’s Hospital. The study included adult female patients (aged > 19 years) scheduled for elective surgery not associated with the neck area. Exclusion criteria included known neck pathologic findings (i.e., thyroid mass, craniofacial deformity, and a history of laryngeal disease), history of neck surgery other than thyroid surgery, extreme obesity (body mass index [BMI] > 35 kg/m^2^), emergency surgery, American Society of Anesthesiologists (ASA) class ≥ III, and refusal to participate in this study. Six patients were excluded because of a history of cervical spine surgery (*n* = 4) or refusal to be involved in this study (*n* = 2). Therefore, 80 adult patients were ultimately analyzed (Figure 1).

The female patients were classified into two groups: a control group (without history of thyroid surgery; *n* = 40) and an experimental group (with history of thyroid surgery; *n* = 40).

### 2.3. Identification of the CTM by Palpation and Sonographic Confirmation in the Operating Room

In the operating room, the patients were placed in a supine neutral position before induction of general anesthesia, and one assessor anesthesiologist (who had been certified in airway management for ten years in operating room and intensive care unit, but was not further involved in the study process and never participated as one of the evaluators to avoid bias in the evaluation) conducted a visual inspection and palpation of the neck to identify the CTM. The caudally located cricoid cartilage was identified after first locating the cricoid cartilage. The space between the two cartilage structures was identified as the CTM and marked (on the skin) with a water-based pen. To ensure that the assessor could conduct the palpation accurately and thoroughly without being rushed, we did not impose any time or attempt limits on the CTM palpation measurement.

While the assessor anesthesiologist palpated the CTM, the evaluators waited outside the operating room to ensure an unbiased evaluation process. After marking it, the assessor anesthesiologist exited of the operating room, and the evaluators came in and confirmed the location of CMT by ultrasound independently, ensuring that each assessment was conducted without influence from the previous evaluations.

Three evaluator anesthesiologists individually assessed whether CTM palpation was successful using bedside ultrasonography [3,4]. The evaluator anesthesiologists, each with a decade of airway management experience in both the operating room and the intensive care unit, served as evaluators. However, they did not engage further in the study’s process nor did they take part as assessors, ensuring the avoidance of any bias in the evaluations. When two or more evaluators confirmed the accuracy of surface landmark palpation for CTM identification, it was defined as successful CTM palpation and marking (Supplemental File S1). A portable ultrasound machine with a 4–12 MHz linear probe (Philips EPIQ 7 Ultrasound Machine; Philips, Amsterdam, The Netherlands) was used for identification. The transducer was transversely placed at the level of the thyroid cartilage, and the thyroid cartilage was identified as a hyperechoic structure. The transducer was moved caudally, and the hyperechoic line was identified; this was the air-tissue border of the CTM. The transducer was moved caudally until the cricoid cartilage was seen, as a black “C”. Finally, the transducer was moved in the cephalad direction and the CTM was identified [1]. When the mark of CTM was present in the probe area, it was evaluated as correct (Supplemental File S2).

### 2.4. Primary Outcome

We compared the successful identification rates of the CTM palpation method between two groups of female patients: those with a history of thyroid surgery and those without a history of thyroid surgery.

### 2.5. Perioperative Factors

The perioperative factors of interest included age, sex, BMI, comorbidities (e.g., diabetes mellitus or hypertension), and laboratory parameters (i.e., hemoglobin, platelet count, and alanine aminotransferase).

### 2.6. Statistical Analyses

The minimum sample size required to detect a difference in the success rate of identification of the CTM between two groups was calculated. Based on preliminary data at our hospital (unpublished), the identification success rates in those without a history of thyroid surgery (*n* = 10) and with a history of thyroid surgery (*n* = 10) were 80% and 50%, respectively. Therefore, a minimum sample size of 39 patients in each group was required (α = 0.05, power = 0.8). Assuming a drop-out rate of 10%, 86 patients were included.

We compared the CTM identification success rate and peri-operative factors between two groups using the Mann–Whitney U test and the chi-square test, as appropriate. Continuous data are presented as medians and interquartile ranges (IQRs), and categorical data as numbers and proportions. In all analyses, *p* < 0.05 was taken to indicate statistical significance. The predictive accuracy for CTM palpation was evaluated with the area under the receiver operating characteristic (ROC) curve (AUC). Statistical analyses were performed using SPSS for Windows (ver. 24.0; IBM Corp., Chicago, IL, USA), R (ver. 2.10.1; R Foundation for Statistical Computing, Vienna, Austria) and MedCalc for Windows software (ver. 11.0; MedCalc, Ostend, Belgium).

## 3. Results

The participants in this study had an average age of 45.9 years with a standard deviation of 13.2 years, and an average BMI of 24.7 with a standard deviation of 4.1 kg/m^2^. Within the experimental group, lobectomy was the most common procedure, performed on 67.5% of the patients (*n* = 27). Total thyroidectomy was conducted in 27.5% of cases (*n* = 11), while isthmusectomy was reported in 5% of the patient cohort (*n* = 2). The median weight of the resected thyroid tissue was 12 g, with an interquartile range from 7.6 to 20.8 g. Additionally, diabetes mellitus and hypertension were present in the patient population at rates of 5% (*n* = 4) and 20% (*n* = 16), respectively.

No significant demographic differences were noted between the control and experimental groups, as shown in Table 1. The control group demonstrated a significantly higher success rate in CTM palpation identification at 90%, compared to 42.5% in the experimental group (*p* < 0.001, Table 2). In female patients without a history of thyroid surgery, CTM palpation had a high sensitivity of 90% and a specificity of 57.5% based on 36 true positives, 17 false positives, 23 true negatives, and 4 false negatives, indicating a strong ability to identify true CTM presence. Conversely, in female patients with a history of thyroid surgery, the sensitivity was notably lower at 42.5%, with a specificity of 10%, as calculated from 17 true positives, 36 false positives, 4 true negatives, and 23 false negatives, suggesting a diminished ability to correctly identify CTM.

## 4. Discussion

This study meticulously examines the implications of prior thyroid surgery on the palpation accuracy of the CTM among female patients. With a detailed exploration into the effects of various thyroid surgical procedures—including lobectomies, total thyroidectomies, and isthmusectomies—the research illuminates how surgical alterations to the neck’s anatomy can complicate the critical task of CTM identification. The findings underscore the challenge posed by post-operative anatomical changes, revealing a significant decrease in palpation accuracy among patients with a history of thyroid surgery compared to those without such interventions. This research advocates for an enhanced focus on tailored airway management training that takes into consideration the nuanced difficulties encountered in patients with altered neck anatomy due to thyroid surgery, aiming to elevate the standard of care and patient safety in emergency airway management scenarios.

Various factors influence the direct and accurate palpation of the CTM, with a wide range of success rates reported depending on the study design. Conventionally, the CTM is identified by palpation between the cricoid and thyroid cartilages, but this traditional landmark technique has been found to be inaccurate [9]. Studies focusing on CTM identification have indicated that the digital palpation (DP) method may not consistently achieve accurate and uniform identification of the CTM [10]. Several factors, including sex, pregnancy, obesity, facial trauma, and other neck pathologies, can influence the misidentification of the CTM through DP [3,8]. Contrastingly, Elliot et al. [11] reported that the delineation of a cricothyrotomy entry point was not significantly affected by patient characteristics such as weight, height, BMI, neck circumference, or CTM dimensions. You-Ten et al. [3] found that DP performed by anesthesiologists accurately localized the CTM in 71% (20/28) of non-obese patients compared to only 39% (11/28) of obese patients (*p* = 0.03). Moreover, Aslani et al. [4] demonstrated that the CTM was identified in 24% (10/41) of non-obese vs. 0% (0/15) of obese female patients in the neutral neck position (*p* = 0.048). These findings correlate with difficult airway predictor tools that include anatomical characteristics such as the prominence of the thyroid cartilage, the CTM’s vertical height and width, and the anterior neck thickness [12]. Lee et al. [13] suggested that female patients undergoing non-neck surgery with shorter distances between interincisors (<3 fingerbreadths), hyoid-to-mental distances (<3 fingerbreadths), or thyroid-to-hyoid distances (<2 fingerbreadths) might have a higher risk of failure in terms of accurately palpating the CTM compared to those with longer distances. Additionally, patients identified as having difficult airway variables had higher BMIs compared to those without, despite having similar age distributions. The success rate of CTM palpation was significantly higher in the non-difficult airway group (93%, 28/30) than in the difficult airway group (70%, 21/30). This study, indicating a 90% success rate of CTM palpation, suggests that the inclusion of only female patients, with a small portion of obese patients, to minimize the impact of sex differences on airway anatomy and to measure the impact of thyroid surgery on CTM palpation may have contributed to the observed difference in success rates between studies that included both sexes.

Misidentification of the CTM by palpation occurs frequently in female patients [4]. The CTM identification success rate was lower in female and obese patients (35%) than in non-obese male patients (72%) in a previous study [14]. The thyroid cartilage is an important palpable structure for identifying the CTM [8]; however, because it is less prominent and smaller in females, misidentification of the CTM is more likely than in males. The cricoid cartilage was mistaken for the thyroid cartilage more frequently in female patients (80% vs. 30%, respectively), which may be the cause of CTM misidentification [15]. In addition, the vertical height and maximum width of the CTM are greater in males than in females [16]. Identifying the CTM by palpation is more difficult in patients with difficult neck anatomy than in those with normal neck anatomy. The CTM identification success rate in patients with a neck pathology, including a history of neck surgery, radiation therapy, or a neck mass, was reported as 8% [6]. Jimbo et al. reported that the CTM could not be identified in patients with large cervical hematomas [17]. The success rate of transcricothyroid placement of a cannula was 44% in a model simulating unidentifiable neck anatomy [18]. In this study, we found that the success rate of CTM identification was significantly lower in female patients with a history of thyroid surgery. The surgical trauma from thyroid surgery, including retraction of the musculature and dissection of soft tissue, can alter neck anatomy. Consequently, tissue edema or hematoma formation following surgical trauma may impair the clear identification of the CTM. This issue may be exacerbated in females, who generally have less prominent neck landmarks, making them potentially more susceptible to difficulties in CTM identification due to surgical alterations.

Ultrasound is highly valued in clinical settings for its non-invasiveness, portability, and high accuracy [19], making it an effective tool for delineating neck airway anatomy and aiding in rescue airway management [20]. Nicholls et al. confirmed the accuracy of ultrasound in identifying the CTM in a cadaver study [21], and its efficacy has been further demonstrated in improving CTM identification accuracy in obese women [3]. Moreover, Siddiqui et al. found that ultrasound outperformed palpation in terms of identifying the CTM in patients with indistinct neck anatomy [6]. In our study, we localized the CTM using ultrasound, building upon the methodology of a previous study [1]. However, the use of ultrasound for CTM identification is not without challenges, including potential technical difficulties and reduced visibility in some cases [4]. Acquiring and interpreting ultrasound images accurately requires practice and technical skill [14]. The setup time for ultrasound equipment may pose a delay during urgent procedures like cricothyrotomy. Post-thyroidectomy, surgical exploration becomes critical in instances such as post-operative bleeding. Furthermore, in situations where cricothyroidotomy proves difficult, alternative approaches like needle cricothyroidotomy may be considered. Ultrasound is less effective in environments with subcutaneous air, as it hampers the visualization of anatomical structures beyond the air presence [22]. Although ultrasound’s diagnostic accuracy is generally high and comparable to CT, with reported accuracies ranging from 81% to 100% in various studies [1,6], it can be more variable than CT imaging [23].

Our study faced several limitations. Firstly, among various types of neck surgery, we only included patients with a history of thyroid surgery due to the complex and diverse surgical techniques involved in neck operations. Thyroid surgery is common in women, and typically, the surgical scars are located differently from the CTM, thus minimally impacting measurements. However, focusing solely on thyroid surgery limits our ability to conclusively determine its impact on CTM measurement accuracy in the context of all neck surgeries. Secondly, our study’s non-randomized and unblinded design introduces the risk of selection bias. Nonetheless, considering the high prevalence of thyroid disease in women [24], our findings highlight the importance of heightened caution in airway management for patients with a history of thyroid-related surgery, marking a significant strength of our study. Future clinical research should focus on expanding its scope by exploring novel palpation techniques, creating specialized training modules for healthcare professionals, and conducting comparative analyses of various surgical methods. These initiatives could yield significant advancements in optimizing airway management strategies for patients who have undergone surgery. By addressing these areas, the research could lead to improved outcomes in the identification and handling of the CTM in post-surgical patients, ultimately enhancing patient care and safety during airway management procedures.

## 5. Conclusions

In this study, we discovered that the success rate for identifying the CTM was notably lower in female patients who had undergone thyroid surgery. The alterations in neck anatomy, a result of surgical interventions such as muscle retraction and soft tissue dissection, contributed to this reduced accuracy. This challenge is particularly pronounced in females, whose neck landmarks are generally less distinct compared to their male counterparts, making the identification of CTM post-surgery even more difficult. Consequently, female patients with a history of thyroid surgery may necessitate a more careful and refined approach to ensure accurate CTM palpation. These insights emphasize the need for airway management education and training programs to adapt and include strategies that address the unique challenges faced by this patient demographic, enhancing the efficacy of emergency airway access in clinical settings.

## Figures and Tables

**Table 1:** Comparison of perioperative findings in female patients: control group (no history of thyroid surgery) vs. experimental group (with history of thyroid surgery).

Group	Control	Experimental	*p*
n	40	40	
Age (years)	44 (35–57)	46 (39–54)	0.641
Obesity (BMI ≥ 30 kg/m^2^)	5 (12.5%)	3 (7.5%)	0.456
*History of thyroid surgery type*			-
Total thyroidectomy	-	11 (27.5%)	
Rt. or Lt. lobectomy	-	27 (67.5%)	
Isthmusectomy	-	2 (5%)	
Record of thyroid mass weight (g)	-	12 (7.6–20.8)	-
*Comorbidity*			
Diabetes mellitus	3 (7.5%)	1 (2.5%)	0.305
Hypertension	9 (22.5%)	7 (17.5%)	0.576
*Laboratory variables*			
Hemoglobin (g/dL)	13.8 (13.3–14.6)	13.5 (13.2–13.9)	0.11
WBC count (×10^9^/L)	5.9 (5.4–7.1)	6.0 (4.4–6.8)	0.256
Platelet count (×10^9^/L)	242 (203–258)	251 (222–317)	0.044
AST (U/L)	20 (16–25)	17 (15–23)	0.1
ALT (U/L)	20 (12–23)	14 (11–22)	0.094

**Abbreviations:** BMI, body mass index; WBC, white blood cells; AST, aspartate aminotransferase; ALT, alanine aminotransferase. **Note:** Values are expressed as median (interquartile) and number (proportion).

**Table 2:** Comparison of the cricothyroid membrane identification success rates by palpation in female patients: control group (no history of thyroid surgery) vs. experimental group (with history of thyroid surgery).

Group	Control	Experimental	*p*
*n*	40	40	
***Successful rate***			
Palpation identification	36 (90%)	17 (42.5%)	<0.001

**Note:** Values are expressed as numbers and proportions.

## Data Availability

The data presented in this study are available in insert article.

## Supplementary Materials

The following supporting information can be downloaded at: https://www.mdpi.com/article/10.3390/medicina60030471/s1, Supplemental File S1. Evaluator consensus on cricothyroid membrane palpation accuracy: Control Group (No History of Thyroid Surgery) vs. Experimental Group (With History of Thyroid Surgery). Supplemental File S2. Methodical Assessment for Cricothyroid Membrane Localization. (A) Initial Dermatographic Marking: Extended lines drawn with a water-based dermatographic pen on the patient’s neck to delineate the target area, intended to be detectable throughout the ultrasound scanning process. (B) Ultrasound Application: The placement of the ultrasound transducer adjacent to the dermatographic delineations, with the pen’s indelible lines clearly observable on both flanks of the probe, facilitating precise and consistent evaluations by the assessors.

## Abbreviations

ASA, American Society of Anesthesiologists; cricothyroid membrane, CTM; computed tomography, CT; eFONA, emergency front of neck access; IQR, interquartile range.
