# Supplementary material for: The Effect of Thyroid Surgery on the Accuracy of Palpation-Based Cricothyroid Membrane Identification in Female Patients: A Prospective Observational Cohort Study
Source: Medicina (Kaunas). 2024 Mar 13;60(3):471. doi: 10.3390/medicina60030471 (PMC10971964; doi:10.3390/medicina60030471)
Supplement: Supplementary file 1 [file medicina-60-00471-s001.zip › medicina-2888799-supplementary.pdf]

Supplemental File S1. Evaluator consensus on cricothyroid membrane palpation accuracy: Control Group (No History of Thyroid Surgery) *vs*. Experimental Group (With History of Thyroid Surgery).

**Group**	**Control**	**Experimental**	***p***
n	**40**	**40**	
Agreement status			0.775
Full agreement	33 (82.5%)	32 (80.0%)	
Majority agreement	7 (17.5%)	8 (20.0%)	

Full agreement: all three evaluators agreed on the outcome.

Majority agreement: Two of the three evaluators agreed on the outcome.

Note: Values are expressed as numbers and proportions.

Supplemental File S2

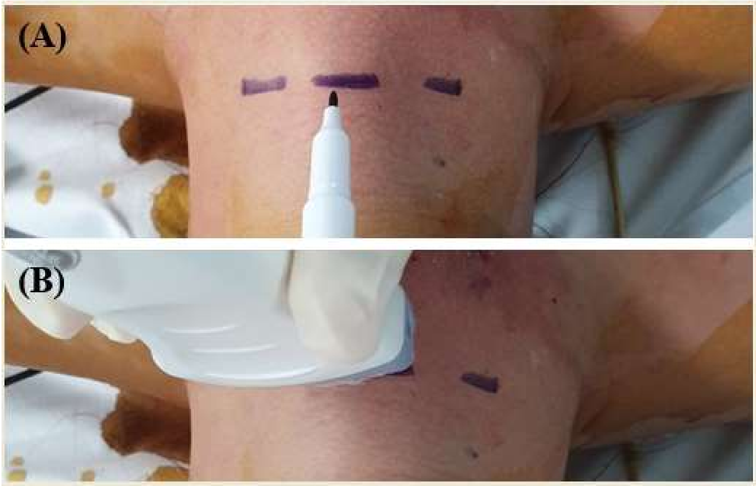

Methodical Assessment for Cricothyroid Membrane Localization. (A) Initial Dermatographic Marking: Extended lines drawn with a water-based dermatographic pen on the patient's neck to delineate the target area, intended to be detectable throughout the ultrasound scanning process. (B) Ultrasound Application: The placement of the ultrasound transducer adjacent to the dermatographic delineations, with the pen's indelible lines clearly observable on both flanks of the probe, facilitating precise and consistent evaluations by the assessors.
